# Facilitators and barriers to implementing the Diabetes Prevention Program in rural church settings: A qualitative study using the Consolidated Framework for Implementation Research

**DOI:** 10.1111/jrh.12888

**Published:** 2024-10-13

**Authors:** Smita Rawal, Caleb A. Snead, Frantz D. Soiro, Jeffery Lawrence, Brian M. Rivers, Henry N. Young

**Affiliations:** ^1^ Department of Clinical and Administrative Pharmacy College of Pharmacy, University of Georgia Athens Georgia USA; ^2^ Department of Health Promotion and Behavior College of Public Health, University of Georgia Athens Georgia USA; ^3^ Interdenominational Ministerial Alliance Georgia USA; ^4^ Department of Community Health and Preventive Medicine Cancer Health Equity Institute Morehouse School of Medicine Atlanta Georgia USA

**Keywords:** CFIR, church, Diabetes Prevention Program, qualitative, rural

## Abstract

**Purpose:**

The CDC's Diabetes Prevention Program (DPP) is an effective lifestyle intervention to prevent type 2 diabetes (T2D). However, DPP implementation in rural areas is limited. This study sought to address this gap by implementing DPP in rural church settings through a community–academic partnership and identifying implementation facilitators and barriers.

**Methods:**

This was a cross‐sectional qualitative study. Semistructured interviews guided by the Consolidated Framework for Implementation Research (CFIR) assessed church leaders' and lifestyle coaches' perceptions of implementing DPP in rural churches. Thematic analysis was used to identify key themes through an inductive approach; then, these emergent themes were deductively linked to CFIR constructs. COREQ guidelines were used to report study findings.

**Findings:**

Twenty‐five stakeholders participated. Facilitators to implementing DPP included its evidence‐based effectiveness in preventing T2D, as well as support from the academic partner in terms of funding, training, and communication. Additionally, DPP's alignment with community needs, along with the active engagement of pastors in participant recruitment, supported implementation. Several barriers hindered DPP implementation, including transportation and childcare issues, as well as program participants' medical conditions/disabilities limiting their participation. Furthermore, rural residents' reluctance to adopt lifestyle changes and loyalty to family churches posed challenges to their engagement in DPP.

**Conclusions:**

This study identified contextual factors influencing DPP implementation in rural communities. Findings highlight the importance of tailored strategies that leverage facilitators while proactively addressing barriers, including rural residents' reluctance to attend programs outside their church, resistance to lifestyle changes, and transportation issues to ensure successful DPP implementation in rural areas.

## INTRODUCTION

Type 2 diabetes (T2D) is one of the leading causes of morbidity and mortality, affecting over 30 million Americans.^1^ The Centers for Disease Control and Prevention (CDC)’s Diabetes Prevention Program (DPP) reduces the risk of T2D through weight loss and increased physical activity.^2^ Studies demonstrate the effectiveness of DPP, with a 58% reduction in T2D cases and an average weight loss of 5–7%.^3^, ^4^, ^5^ It is widely adopted across diverse settings (6–9), including rural areas.^6^, ^7^ However, research indicates a disparity in DPP access, with only 14.6% of rural counties having access compared to 48.4% of urban counties.^8^ This underscores the need for expanding DPP access in rural regions, where T2D prevalence is 16% higher and T2D‐related hospital mortality rate is 20% greater than in urban areas.^9^, ^10^, ^11^, ^12^

Many rural and underserved areas lack diabetes prevention resources due to a shortage of providers and inadequate infrastructure for primary prevention.^8^, ^13^, ^14^ Translating the DPP into these communities through community–academic partnerships (CAPs), particularly by utilizing churches, offers a promising approach to prevent T2D. Churches, being central components of rural communities and accessible to underserved populations, provide opportunities for forming partnerships and implementing evidence‐based programs.^15^, ^16^, ^17^, ^18^ Successful program implementation entails evaluating factors that influence the process, as suggested in the dissemination and implementation (D&I) literature.^19^ Existing studies on factors influencing DPP implementation predominantly focus on medical settings, community centers (YMCAs and senior centers), and the involvement of extension agents.^20^, ^21^, ^22^, ^23^, ^24^, ^25^, ^26^ These studies found barriers, including recruitment issues, CDC recognition processes, data management, expenses, and participant time constraints.^20^, ^21^, ^22^, ^23^, ^24^, ^25^, ^26^ However, less is known about context‐specific facilitators and barriers to implementing DPP through CAPs in rural church settings. Furthermore, existing studies have predominantly conducted post‐hoc analyses, leaving the exploration of factors during the active implementation phase largely unexplored. This study aimed to fill these gaps by conducting a formative evaluation of DPP implementation during the active phase to assess stakeholder perceptions and identify facilitators and barriers.^27^ Determinant frameworks, such as the Consolidated Framework for Implementation Research (CFIR), are useful for identifying factors influencing program implementation.^28^ The CFIR includes 39 constructs across five domains: intervention characteristics, outer setting, inner setting, characteristics of individuals, and process.^28^ The objective of this study was to utilize the CFIR as a guiding framework to identify facilitators and barriers to implementing the DPP within rural church settings and provide recommendations for future implementation.

## METHODS

### Study design

This was a cross‐sectional qualitative study examining the perceptions of stakeholders, including church leaders and lifestyle coaches, about implementing the DPP in rural church settings. The study was approved by the University of Georgia Institutional Review Board (PROJECT00007093). Reporting followed the Consolidated Criteria for Reporting Qualitative Research (COREQ) guidelines (Appendix A).^29^

### Program description and study setting

The University of Georgia research team, in collaboration with the UGA Archway Partnership (a unit of UGA Public Service and Outreach), undertook a community–academic partnership (CAP) to implement the DPP in rural Georgia. The CAP, entitled “*Fishers of Men*,” is a collaborative initiative between academic institutions and a coalition of churches located across rural Georgia that is focused on addressing health disparities in underserved communities.^30^ In February 2023, the academic team traveled with church leaders in rural Georgia to provide insights into the program and the DPP implementation process. By July 2023, 20 churches had received funding, submitted the Diabetes Prevention Recognition Program (DPRP) application, and committed to adopting the program. In May–August, 33 individuals from these churches attended a 3‐day CDC‐approved lifestyle coach training offered by Emory University's Diabetes Training and Technical Assistance Center (DTTAC). This training covered DPP facilitation techniques, data reporting, and standards for maintaining CDC recognition.

**FIGURE 1:**
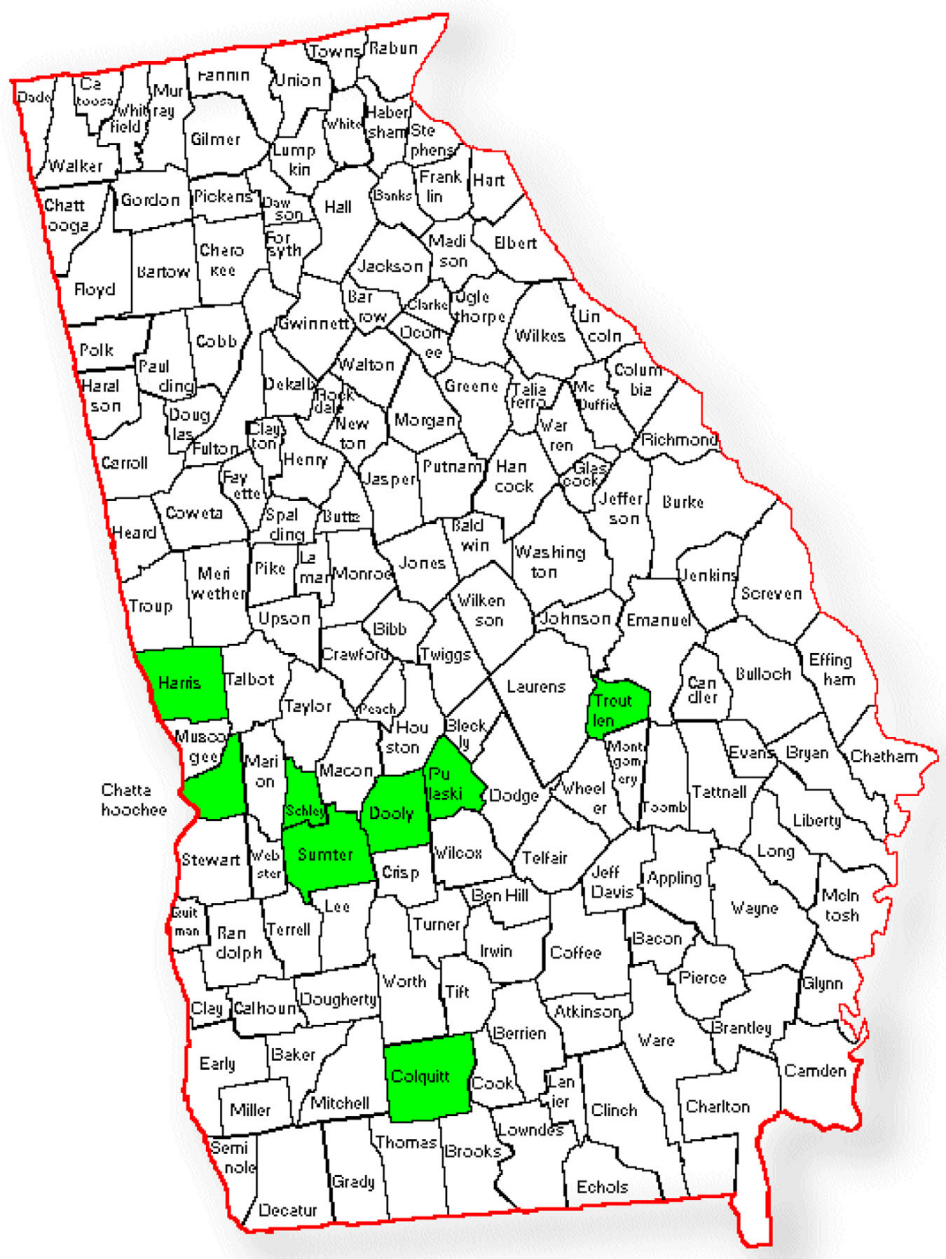
DPP implementation in church settings across 8 rural Georgia counties (Chattahoochee, Colquitt, Dooly, Harris, Pulaski, Schley, Sumter, and Treutlen).

Following the training, the academic partner conducted field visits and bimonthly Zoom meetings to provide technical assistance for lifestyle coaches and church leaders. These meetings provided updates on participant recruitment and program delivery, while also facilitating discussions on the successes and challenges in implementation and engagement. Although the initial recruitment goal was 10 participants per church, totaling 200 participants, churches were encouraged to recruit 15–20 participants each to account for potential dropouts. Recruitment strategies included newspaper advertisements, Facebook announcements on church pages, and word‐of‐mouth. The program ultimately recruited 245 rural participants, averaging 10–15 per church. The DPP implementation commenced in June 2023, involving 20 churches across eight counties in Georgia (see Figure 1) designated as rural by the Georgia Department of Community Health or with a Rural‐Urban Commuting Area code of 6 or higher.^31^, ^32^

### Study participants, recruitment, and data collection

A purposive sample of 30 stakeholders (church leaders/pastors and lifestyle coaches) involved in DPP implementation were invited via telephone and email in October 2023 to participate in the qualitative interview.

Semistructured guides based on CFIR directed the interview process (see Appendix B). Initial interview guides were adapted from sample questions available at http://cfirguide.org/ and a previous study.^33^ Questions were tailored to gather specific information about participants' perceptions/experiences of implementing DPP in rural church settings. Face validity was established by pretesting the interview guide with two participants and two research experts. The guide and process were pilot‐tested with two participants, and probing questions were refined based on feedback. Pretesting and pilot‐testing interviews were not included in the final analyses.

Questionnaires and consent forms were emailed to participants prior to interviews. Two research team members, a postdoctoral researcher with qualitative research experience (S. R.) and a graduate student trained in public health (C.A.S.), conducted one‐to‐one interviews via Zoom between October 2023 and January 2024. Only the interviewer and participant were present during the interview. Interviewers were cautious not to contradict or bias the interviewees' responses. Enrollment and interviews continued until data saturation was achieved. Data saturation occurred when responses became repetitive, and no new themes emerged.^34^ All interviews were audio‐recorded and transcribed verbatim. Transcripts were deidentified to ensure participants’ confidentiality.

### Data analysis

Interview transcripts were coded by thematic analysis.^35^ Themes were identified both inductively, based on interview content, and deductively using the CFIR.^28^ Two research team members (S.R. and C.A.S.) independently coded one interview transcript and then met as a team to reconcile any coding differences.^35^, ^36^ This process was repeated with a second transcript to finalize the codebook. Subsequent transcripts were coded independently by two researchers (S.R. and C.A.S.). Any coding discrepancies/disagreements were resolved through discussions with the faculty investigator experienced in qualitative research (H.N.Y.). Finally, S.R. abstracted all codes into themes, aligned them with CFIR constructs, and created a summary table compiling the findings. The importance of themes identified in qualitative analysis is not necessarily related to the frequency of coding. As a result, expressions such as “all” or “most participants” were used to depict common themes, while “several” or “one participant” indicated minor themes. Participant quotes were included to illustrate identified themes.^37^

### Study's rigor and trustworthiness

The study ensured rigor and trustworthiness by adhering to Lincoln and Guba's four criteria: credibility, confirmability, dependability, and transferability.^38^ Credibility was established through triangulation, member checking, and peer debriefing.^39^, ^40^ Triangulation of sources involved recruiting stakeholders in DPP implementation, including pastors and lifestyle coaches. Investigator triangulation was achieved with two team members (S.R. and C.A.S.) conducting independent data analysis. Member checking was conducted by sharing interview transcripts with participants and seeking their feedback. Peer debriefing included presenting the methodology, interview transcripts, and findings to a faculty member experienced in qualitative research and rural community health practice who had no involvement in the study, thus providing an objective review. Team members engaged in reflexivity to acknowledge and manage personal biases, thereby ensuring confirmability. Dependability was maintained through a detailed audit trail, which included coding frameworks, iterations, and analytic memos.^38^ Transferability was enhanced by including “thick” descriptive quotes, enabling other researchers to understand the relevance of these findings to their own contexts.^37^

## RESULTS

A total of 30 stakeholders (church leaders/pastors and lifestyle coaches) were invited for interviews. Two declined, and another two withdrew for personal reasons. Recruitment occurred on a rolling basis over 4 months, with interviews conducted in batches of three and analyzed iteratively. Recurring themes became noticeable after the 21st interview, and by the 25th interview, no novel themes/sub‐themes emerged, indicating data saturation.

Twenty‐five stakeholders participated, comprising 15 lifestyle coaches and 10 pastors. The mean interview duration was 64 min (SD = 10.8). All participants identified as African American (100%) with some college/college degrees (100%) and had a mean age of 60.7 years (SD = 9.24) (see Table 1).

**TABLE 1:** Demographic characteristics of interview participants (*N* = 25).

Demographic characteristics	*N* (%)
Age (mean, SD)	60.7 (9.2)
Race	
African American	25 (100)
Gender	
Female	14 (56)
Male	11 (44)
Education	
Some college	5 (20)
College degree	20 (80)

Themes obtained from interviews describing facilitators and barriers to implementing DPP were categorized into five CFIR domains: intervention characteristics, outer setting, inner setting, characteristics of individuals, and process of implementation. Facilitators were identified across all five CFIR domains, whereas barriers emerged within the “outer” and “inner setting” domains. These themes, matched to CFIR constructs and supported by illustrative verbatim quotes, are summarized in Table 2 and described in detail below.

**TABLE 2:** Facilitators and barriers to implementing the DPP in rural church settings based on CFIR.

CFIR domains and constructs	Themes	Representative quotes
**I. CFIR‐based facilitators**		
**Intervention characteristics**		
**Intervention Source**	**Involvement of reputed external sources**	“So my hats off to all that were in the trenches and did the groundwork [CDC]. Now that you [UGA] brought it here, you've made it so easy for people to follow…For churches interested in DPP, one of the first things is to make sure that this is something that you're interested in doing. Have credible partnerships and resources to help you do it. If you find those people and resources, you've got a formula for success.” (P2)
**Evidence Strength & Quality**	**Practicality and Effectiveness of the program**	“I like the fact that the CDC program is so structured. And it's a very practical program, and it's proven, you know, it's something that's been proven to be beneficial for preventing diabetes.” (P6)
**Design Quality & Packaging**	**Ease of using guidebooks for program facilitation**	“The easiest part about DPP is using the guidebook information. Everything is done…You even have the guide tell you. Okay, do this, say this to participants, or make a comment here. It's really, really detailed.” (P7)
**Outer setting**		
**Patient needs & resources**	**DPP's fit with community needs**	“We have many community members who are at‐risk or already diagnosed with diabetes. Bringing this program to them is helpful. It helps everyone, also those already diagnosed, to improve their diet and physical fitness.” (LC6)
**Inner setting**		
**Compatibility**	**DPP's alignment with churches’ mission**	“This program fits really like a glove with our church. We have large connectivity and we're community‐oriented, providing food, clothing, shelter, and wellness. Therefore, it really fits well with our church and mission due to our strong community presence…So, my recommendation is to use this opportunity and expand in rural Georgia.” (P9)
**Communications**	**Effective communication strategies**	“We haven't had any issues communicating. We've had training classes; you made visits and set up meetings every other Saturday on Zoom. I've gotten different emails from you, the CDC. So all that has helped with information that I take back to our sessions to help.” (LC6)
**Readiness for implementation**		
**a. Leadership Engagement**	**Leadership's positive influence on recruitment/participation**	“The pastor was really on it and trying to advertise in the community. I feel that because the pastor took a great interest in it, people had no problem joining. If the pastor's happy and enthusiastic about program, they're coming, and they're gonna be happy about it, too. And that's just the phenomenon…The pastor plays a big role in bringing people…For future churches, I recommend that their pastors show interest and advertise to bring people.” (LC2)
**b. Available Resources**	**Funding, space, and training support**	“Funding was a major thing in getting us started and having what we needed, lifestyle coach training, weight scales, incentives, and everything else…As we continue, you know, it's an asset. Moving forward, I think funding is the major thing, and it's essential for program continuation and expansion into other communities.” (P5) “Training gave us the tools to follow the steps, to be able to get started within the church…The size of our sanctuary, the size of our fellowship hall is pretty large, so you can move around.” (LC8)
**Characteristics of individuals**		
**Knowledge and beliefs about the intervention**	**Increased program awareness through education**	“We weren't aware of this program. We are aware now, after UGA's efforts…a program of this magnitude, I wasn't exposed to it, and I will say probably 99.9% of the church members weren't exposed to it. We are a rural area, and things like this weren't offered…So, your outreach has benefited us and will be helpful for other churches.” (P3)
**Self‐efficacy**	**Lifestyle coaches’ personal growth and adaptability**	“After training, meetings, and sharing ideas, I'm learning more about myself and working with people in the sessions…Continuing training, sharing successes and problems, and keeping that going, I think that'll be helpful.” (LC9) “Some days that participant cannot attend due to the experiences with this CP [cerebral palsy], but then I make a home visit.” (LC5)
**Process**		
**Planning**	**Structured program plan**	“We have a clear plan…it's on a notebook, and also on computer; how the sessions go, how funds will be transmitted every week.” (P3)
**Reflecting & Evaluating**	**Continuous reflection and communication**	“This is what's going on. These are the benefits. These are the goals [healthy eating and achieving weekly physical activity targets]. We're having constant dialogue, communication in terms of making this really work, meeting the CDC goals.” (P2)
**II. CFIR‐based barriers**		
**Outer setting**		
**Patient needs & resources**	**Accessibility challenges due to rurality**	“I would say it would be the transportation. Some of the participants may have schooling or time restraints, childcare. That's what you deal with a lot here [in rural areas].” (LC8) “In rural areas, we've elderly people with medical conditions. One of the participants is disabled with cerebral palsy… Some days that participant cannot attend due to the experiences with this CP [cerebral palsy], but then I make a home visit…So, I feel medical issues would be the biggest constraint if they aren't able to finish. We can step up; do things like call or home visits to help them catch up.” (LC5) “The most difficult part for our church is we are in a rural area. People live a great distance from the church…We had to tell people who could get here to bring others along the way. And, we decided we will have DPP on Wednesday, Bible study night. And that seems to be working out pretty good right now. So, definitely, transportation, giving people rides, and scheduling on Bible study night helped us with participation and can help other rural churches.” (P3)
**Inner setting**		
**Culture**	**Cultural dynamics in rural communities**	
	**a. Rural residents' reluctance to join programs outside of their family church**	“I'm a bit surprised that we didn't get an overwhelming number of participants because we've been so community‐oriented. As far as the church is concerned…people are loyal and show loyalty to their own, their family church. Yeah, we see that all the time.”(P9)
	**b. Resistance to change in rural communities**	“Well, people are set in their ways, in terms of how they eat, what they eat, when they eat, those types of things. In rural Georgia, people have a different mentality about health and wellness. So we've had to fight, show people that this works. By the end, these churches and communities will have witnesses, people who have benefited from the program that can say, Yes, I did the program for a year. It was fun, engaging, and it works.” (P2)

Abbreviations: CFIR, Consolidated Framework for Implementation Research; LC, lifestyle coach; P, pastor.

### CFIR‐based facilitators

#### Intervention characteristics

The first CFIR domain is related to the characteristics of the intervention being implemented. Study participants reported three key attributes of the DPP that facilitated its implementation: its intervention source, strength and quality of evidence, and design quality and packaging.

#### Intervention source: Involvement of reputed external sources

Intervention source refers to the perceived advantage of the intervention being developed, either internally (within the organization) or externally (from an outside source). All participants attributed successful DPP implementation to the involvement of reputable and credible external sources, such as the CDC and the academic partner. They advised future sites to cultivate a genuine interest in implementing the DPP, establish partnerships with reputable entities to enhance program credibility and leverage available resources/expertise.

*So my hats off to all that were in the trenches and did the groundwork [CDC]. Now that you [UGA] brought it here, you've made it so easy for people to follow…For churches interested in DPP, one of the first things is to make sure that this is something that you're interested in doing. Have credible partnerships and resources to help you do it. If you find those people and resources, you've got a formula for success. (P2)*

#### Evidence strength and quality: Practicality and effectiveness of the program

Evidence strength and quality pertain to stakeholders’ belief in the intervention's ability to achieve desired outcomes. All participants praised the practicality and effectiveness of the DPP. They commended its structured and practical approach to delivering sessions and its evidence‐based effectiveness in preventing T2D.

*I like the fact that the CDC program is so structured. And it's a very practical program, and it's proven, you know, it's something that's been proven to be beneficial for preventing diabetes. (P6)*

#### Design quality and packaging: Ease of using guidebooks for program facilitation

Design quality and packaging indicate how well the intervention is organized and presented. Most participants expressed ease in conducting the DPP sessions using the manual (guidebooks). They described the guidebooks as simple and providing clear instructions for facilitating the sessions.

*The easiest part about DPP is using the guidebook information. Everything is done…You even have the guide tell you. Okay, do this, say this to participants, or make a comment here. It's really, really detailed. (P7)*

### Outer setting

The outer setting domain encompasses relationships an organization has within the larger economic and social context in which it resides. In this domain, the construct of patient needs and resources facilitated implementation.

#### Patient needs and resources: DPP's fit with community needs

This construct evaluates the extent to which patient needs, as well as barriers and facilitators to meet those needs, are known and prioritized by the implementers. Participants reported that rising rates of T2D and increasing demand for lifestyle change programs in their communities drove the implementation of DPP.

*We have many community members who are at‐risk or already diagnosed with diabetes. Bringing this program to them is helpful. It helps everyone, also those already diagnosed, to improve their diet and physical fitness. (LC6)*

### Inner setting

The inner setting refers to the features of the organization/context where the intervention is implemented. Facilitators included compatibility, communications, and readiness for DPP implementation.

#### Compatibility: DPP's alignment with churches’ mission

Compatibility refers to how well the intervention fits within the organization's existing services and workflows. All participants described the DPP's perfect alignment with their church's mission to address community needs and improve health outcomes. One pastor suggested leveraging the church's established community presence to expand the DPP across rural Georgia.

*This program fits really like a glove with our church. We have large connectivity and we're community‐oriented, providing food, clothing, shelter, and wellness. Therefore, it really fits well with our church and mission due to our strong community presence…So, my recommendation is to use this opportunity and expand in rural Georgia. (P9)*

#### Communications: Effective communication strategies

Communications encompass the structures and practices for sharing information. Participants highlighted efficient and timely information sharing, attributed to diverse strategies by the academic partner—including regular emails, phone calls, field visits, and bimonthly virtual meetings. They expressed that these diverse communication strategies helped them stay updated with information and utilize it effectively in their sessions.

*We haven't had any issues communicating. We've had training classes; you made visits and set up meetings every other Saturday on Zoom. I've gotten different emails from you, the CDC. So all that has helped with information that I take back to our sessions to help. (LC6)*

#### Readiness for implementation

Readiness for implementation, including leadership engagement and available resources, indicates the organizational commitment to implementing an intervention.

#### Leadership engagement: Leadership's positive influence on recruitment/participation

All participants attributed their church's readiness for implementing DPP to their pastors' proactive recruitment efforts and supportive environment. They elaborated that pastors demonstrating genuine interest and actively promoting the program were important for community buy‐in. Participants recommended that pastors at future DPP sites adopt similar approaches to garner community support.

*The pastor was really on it and trying to advertise in the community. I feel that because the pastor took a great interest in it, people had no problem joining. If the pastor's happy and enthusiastic about program, they're coming, and they're gonna be happy about it, too. And that's just the phenomenon…The pastor plays a big role in bringing people…For future churches, I recommend that their pastors show interest and advertise to bring people. (LC2)*

#### Available resources: Funding, space, and training support

Most participants expressed that the availability of resources, including funding, space for DPP sessions, and lifestyle coach training supported DPP implementation. They described using funds to acquire equipment like weighing scales and to provide incentives for program participants. Participants also stressed the necessity of funding for maintaining program continuity and to facilitate potential expansions to other communities.

*Funding was a major thing in getting us started and having what we needed, lifestyle coach training, weight scales, incentives, and everything else…As we continue, you know, it's an asset. Moving forward, I think funding is the major thing, and it's essential for program continuation and expansion into other communities. (P5)*

One participant described that the lifestyle coach training enabled them to initiate the program effectively within their church. They also highlighted the ample physical space available in their sanctuary and fellowship hall, which comfortably accommodated the DPP activities.

*Training gave us the tools to follow the steps, to be able to get started within the church…The size of our sanctuary, the size of our fellowship hall is pretty large, so you can move around. (LC8)*

### Characteristics of individuals

This CFIR domain is related to the individuals involved in intervention implementation. Two prominent themes emerged as DPP facilitators: knowledge and beliefs about intervention and self‐efficacy.

#### Knowledge and beliefs about intervention: Increased program awareness through education

Knowledge and beliefs about the intervention refer to individuals’ attitudes and their familiarity with the intervention. Most participants acknowledged initially lacking awareness of the DPP due to limited program offerings in rural areas. However, they expressed positive attitudes once informed, crediting their improved understanding to educational efforts led by the academic partner. Participants emphasized that these outreach efforts benefited their community and held promise for other rural areas planning to implement the DPP.

*We weren't aware of this program. We are aware now, after UGA's efforts…a program of this magnitude, I wasn't exposed to it, and I will say probably 99.9% of the church members weren't exposed to it. We are a rural area, and things like this weren't offered…So, your outreach has benefited us and will be helpful for other churches. (P3)*

#### Self‐efficacy: Lifestyle coaches’ personal growth and adaptability

Self‐efficacy is defined as an individual's belief in their own capabilities to execute courses of action to achieve implementation goals.^41^ Participants discussed the importance of ongoing training, frequent meetings, and sharing experiences to further enhance their facilitating skills and ensure sustained success in delivering DPP sessions.

*After training, meetings, and sharing ideas, I'm learning more about myself and working with people in the sessions…Continuing training, sharing successes and problems, and keeping that going, I think that'll be helpful. (LC9)*

Several participants also reported that training and technical support meetings boosted coaches’ confidence and enabled them to adapt strategies to meet DPP participants' needs. For example, some coaches made home visits for participants unable to attend sessions regularly due to health conditions.

*Some days that participant cannot attend due to the experiences with this CP [cerebral palsy], but then I make a home visit. (LC5)*

### Process

The final CFIR domain includes process‐related factors. Within this domain, key elements facilitating DPP implementation included planning and reflective evaluation of the program.

### Planning: Structured program plan

Planning is defined as the degree to which implementation tasks are prepared in advance. Participants described their strategy for the year‐long DPP and explained that they documented plans both in notebooks and computers. Most followed a structured approach, outlining DPP schedules for weekly and monthly sessions, including budgeting for supplies/incentives.

*We have a clear plan…it's on a notebook, and also on computer; how the sessions go, how funds will be transmitted every week. (P3)*

### Reflecting and evaluating: Continuous reflection and communication

Goals and feedback involve quantitative/qualitative feedback about the progress of implementation. Participants felt that reflecting on and evaluating DPP goals, which involved constant communication among coaches and program participants about adopting healthy eating habits and achieving weekly physical activity targets, facilitated implementation.

*This is what's going on. These are the benefits. These are the goals [healthy eating and achieving weekly physical activity targets]. We're having constant dialogue, communication in terms of making this really work, meeting the CDC goals. (P2)*

### CFIR‐based barriers

Barriers to DPP implementation were related to the “patient needs and resources” construct in the “outer setting” and the “culture” construct in the “inner setting” domains.

### Outer setting

#### Patient needs and resources: Accessibility challenges due to rurality

Participants highlighted several patient‐related challenges that hindered DPP recruitment/ retention, including transportation issues, childcare responsibilities, and medical conditions.

Many participants commented that some of their program participants faced difficulties attending sessions due to transportation issues, schooling schedules, or childcare responsibilities.

*I would say it would be the transportation. Some of the participants may have schooling or time restraints, childcare. That's what you deal with a lot here [in rural areas]. (LC8)*

Several participants highlighted that rural areas often have a population comprising elderly individuals with medical conditions/disabilities, which can prevent them from attending DPP sessions. Some lifestyle coaches conducted home visits for individuals with medical conditions to enhance retention. They suggested that future implementers adopt supportive strategies to accommodate participants facing challenges in attending sessions.

*In rural areas, we've elderly people with medical conditions. One of the participants is disabled with cerebral palsy… Some days that participant cannot attend due to the experiences with this CP [cerebral palsy], but then I make a home visit…So, I feel medical issues would be the biggest constraint if they aren't able to finish. We can step up; do things like call or home visits to help them catch up. (LC5)*

Furthermore, several participants shared the additional challenge of being in rural areas where some individuals live considerable distances from churches. Some churches provided transportation assistance and organized carpooling for participants without means of transport or those living far away to address the transportation issue. They also rescheduled their DPP sessions to coincide with days when community members were more likely to attend church activities, such as Bible study nights. They suggested that these efforts could benefit other churches facing similar challenges.

*The most difficult part for our church is we are in a rural area. People live a great distance from the church…We had to tell people who could get here to bring others along the way. And, we decided we will have DPP on Wednesday, Bible study night. And that seems to be working out pretty good right now. So, definitely, transportation, giving people rides, and scheduling on Bible study night helped us with participation and can help other rural churches. (P3)*

### Inner setting

#### Culture: Cultural dynamics in rural communities

Culture refers to the shared values, beliefs, and norms across an implementation setting. Participants discussed how cultural dynamics influence DPP implementation in rural areas. They described that rural communities often exhibit loyalty to their family churches, potentially hindering participation in programs offered by external entities. For example, one pastor expressed surprise at the relatively low participation rates despite the church's extensive community service efforts.

*I'm a bit surprised that we didn't get an overwhelming number of participants because we've been so community‐oriented. As far as the church is concerned…people are loyal and show loyalty to their own, their family church. Yeah, we see that all the time. (P9)*

In addition, participants highlighted “resistance to change” among rural residents, often attributed to entrenched habits such as traditional diets and slow‐paced lifestyles prevalent in rural communities. They suggested addressing these barriers by demonstrating the effectiveness of DPP through participant testimonials to promote program adoption within these communities.

*Well, people are set in their ways, in terms of how they eat, what they eat, when they eat, those types of things. In rural Georgia, people have a different mentality about health and wellness. So we've had to fight, show people that this works. By the end, these churches and communities will have witnesses, people who have benefited from the program that can say, Yes, I did the program for a year. It was fun, engaging, and it works. (P2)*

## DISCUSSION

This study used the CFIR to examine the facilitators and barriers to implementing the DPP in rural church settings. Facilitating factors were identified across all five CFIR domains, with key facilitators predominantly belonging to the “inner setting.” Barriers were identified within the “outer setting” and “inner setting” domains. Identifying these contextual factors provided valuable insights to inform future strategies for implementing the DPP in rural contexts.^42^

In this study, three constructs within the “inner setting”—compatibility, communications, and readiness for implementation (with two sub‐constructs—leadership engagement and available resources), facilitated DPP implementation. These findings align with previous research that highlighted the substantial impact of “inner setting” factors on program implementation.^43^ “Compatibility” was evident with the alignment of DPP with churches’ mission to address community needs and improve health outcomes. Effective “communications” characterized by consistent engagement through various modes and timely feedback among implementers also supported DPP implementation. These findings highlight the importance of aligning evidence‐based intervention with the organizational mission and establishing reliable communication structures and processes for successful DPP implementation.^25^, ^44^, ^45^, ^46^, ^47^

The facilitator role of other “inner setting” constructs—leadership engagement and available resources (including funding and logistics) in DPP implementation is consistent with prior studies.^25^, ^48^, ^49^, ^50^, ^51^, ^52^, ^53^ Leadership engagement in this study went beyond superficial program support and entailed pastors’ proactive involvement in DPP delivery.^48^, ^49^, ^50^, ^51^, ^52^, ^53^ This included pastors gaining a comprehensive understanding of the DPP's process and goals, promoting community buy‐in, facilitating logistical support by providing space and dedicated time for DPP sessions, making clear plans to execute sessions, and achieving DPP goals. These findings suggest that DPP implementation in rural church communities may benefit from interventions targeting leadership engagement, communication, and resource planning (availability of funds and logistics). Targeting these factors may increase implementers’ perception of relative priority and strengthen implementation readiness.^54^

Amidst these facilitators, the “inner setting” also presented barriers to DPP implementation. Rural residents' reluctance to participate in DPP outside of their family church and resistance to changing traditional lifestyle habits constituted a “cultural” barrier within the “inner setting.” This aligns with previous research suggesting that rural residents may hesitate to embrace new programs and adopt new habits due to limited exposure/awareness about available programs.^18^, ^55^, ^56^, ^57^ Addressing this challenge could involve implementing a more gradual DPP educational program, maintaining consistent outreach efforts, providing contingency rewards, or employing patient navigators to guide participants through the process.^18^, ^58^ Additional barriers to DPP accessibility were found within the “outer setting.” These impediments, including lack of childcare or transportation for attending DPP sessions, are inherent to rural areas. Previous studies have identified these logistical barriers as obstacles to implementing diabetes care programs and contributing to disparities in diabetes care access.^8^, ^13^, ^14^ Addressing these barriers early in implementation through tailored strategies is important for advancing health equity in rural communities.^59^, ^60^, ^61^

Several valuable lessons have emerged from this study, where “inner” and “outer setting” characteristics predominantly influenced DPP implementation in rural areas. First, it is important to prioritize community needs and ensure program compatibility when implementing evidence‐based programs, alongside securing adequate resources such as funding and logistics. This ensures the program addresses prevalent health concerns, remains relevant, and is successfully implemented. Second, engaging community leaders and securing buy‐in facilitates participant recruitment and enhances program uptake. Third, cultural norms are important in rural areas where traditions and habits may influence program participation and receptiveness to lifestyle changes. Adapting program delivery to incorporate these cultural nuances may foster community acceptance and participation. Fourth, addressing logistical barriers like lack of transportation/childcare in rural is essential. Implementing proactive measures to overcome these barriers can improve program accessibility and ensure equitable participation. Overall, these lessons underscore the importance of a community‐based participatory approach in program implementation, which may facilitate the uptake and enhance the impact of the DPP in rural communities. Table 3 details recommended steps for future DPP implementation in rural settings.

**TABLE 3:** Steps for future DPP implementation in rural church settings.

Steps	Description	CFIR domain/construct
Prioritize community needs	Assess and align the program with prevalent health concerns and ensure it is compatible with community priorities.	Outer Setting: Patient needs & resources
Ensure program compatibility	Ensure that offering DPP is integrated with the church's current services, activities, and workflows.	Inner Setting: Compatibility
Ensure resource availability	Secure and ready resources such as funding, infrastructure, and training to support DPP implementation.	Inner Setting: Readiness for implementation
Leverage church/community leaders	Engage influential and enthusiastic church/community leaders to facilitate recruitment, enhance program buy‐in, and increase uptake.	Inner Setting: Leadership engagement
Acknowledge cultural norms	Recognize and integrate cultural traditions and habits to improve program receptiveness and participation.	Inner Setting: Culture
Address logistical barriers	Proactively tackle needs/issues, such as transportation and childcare, to improve accessibility and ensure equitable participation.	Outer Setting: Patient needs and resources

Abbreviations: CFIR, Consolidated Framework for Implementation Research; DPP, Diabetes Prevention Program.

### Strengths and limitations

This study contributes to implementation science literature by using the CFIR to identify implementation factors specific to implementing DPP in rural church settings. Rigorous qualitative methods, including investigator triangulation, peer debriefing, member checking, reflexivity, and the inclusion of minor themes, ensure methodological rigor and trustworthiness. The consistency of themes and participant quotes strengthens the credibility of the findings.

While this study used a rigorous approach and addresses a gap in the literature on implementing DPP in rural church settings, several limitations exist. First, the cross‐sectional design and qualitative analysis limit the interpretation of relationships between constructs. Second, qualitative data were gathered solely from DPP implementers, excluding perspectives from DPP participants. Third, findings are based on self‐reported responses, potentially introducing biases such as social desirability and recall bias. Fourth, selection bias may exist as interviewees could differ from those who declined participation. Lastly, data saturation is a theoretical concept, and potential misinterpretation of the saturation point should be acknowledged.

## CONCLUSIONS

This study used a CFIR‐based framework to identify facilitators and barriers to implementing the DPP in rural church settings. Tailored strategies are needed for implementing DPP in rural areas. This involves leveraging available facilitators and addressing barriers, including rural residents' reluctance to engage in programs outside their church, resistance to lifestyle changes, and transportation issues. Future implementation efforts should consider these recommendations, explore scalability across wider rural church settings, and assess effectiveness, impact, and long‐term sustainability.

## CONFLICT OF INTEREST STATEMENT

The authors declare no conflicts of interest.

## PREVIOUS PRESENTATION OF THE WORK

The abstract was presented at the American Pharmacists Association (APhA) Annual Meeting. The meeting was held at the Orange County Convention Center, Orlando, FL, USA, from March 22–25, 2024.

## Supporting information



Supporting Information

Supporting Information
