# Supplementary material for: Facilitators and barriers to implementing the Diabetes Prevention Program in rural church settings: A qualitative study using the Consolidated Framework for Implementation Research
Source: J Rural Health. 2024 Oct 13;41(2):e12888. doi: 10.1111/jrh.12888 (PMC11950418; doi:10.1111/jrh.12888)
Supplement: Supplementary file 2 — Supporting Information [file JRH-41-0-s001.docx]

**Church Leadership/Pastors Interview Guide**

The purpose of this interview is to help us understand factors related to conducting the Diabetes Prevention Program (DPP) sessions in the church setting. During the interview, we will ask you about your opinions of DPP and your experience in carrying out the DPP sessions in your church. Your answers will help us to identify any obstacles and improve them for the future.

| **CFIR domain and constructs** | **Interview Questions** | | | |
| --- | --- | --- | --- | --- |
| Role in Church and the DPP | 1. Can you share your roles and responsibilities in your church and the DPP?   ***Probes***   - How did your role as a church leader help implement DPP at your church? | | | |
| Program Administration | 1. Can you walk us through how the DPP ran at your church site on a day-to-day basis?   ***Probes***   - CDC recognition process and paperwork, finding a lifestyle coach, participant recruitment/ retention, DPP sessions, data management  1. Can you describe your overall experience related to the DPP? In general, how is the program going? | | | |
| **DPP Implementation and Program Characteristics: Let’s talk about the DPP at your church.** | | |  | |
| Planning | 1. Did you have a clearly communicated implementation plan for the DPP? Can you describe the plan you have and how it helped or hindered implementation?   ***Probes***   - Was it a formal plan? Was it written down somewhere? And was it updated? - What would you suggest for future church sites when planning the DPP implementation? | | | |
| Compatibility | 1. Did the program fit with the church’s mission and the needs of the community? | | | |
| Evidence Strength and Quality | 1. Before starting the program, what did you know about how well DPP works? (For example, how well does the program help with things like eating healthier, losing weight, or lowering HbA1c levels?   ***Probes***   - Where did you get this information from? (e.g., internet, CDC guidelines, UGA research team presentations, your primary care physician, community pharmacy) | | | |
| Complexity | 1. What was the most difficult part of carrying out the DPP in your church/community? 2. What was the easiest part of implementing the DPP? | | | |
| Adaptability | 1. Did you need to make any changes to implement the DPP in your church? If so, what changes were made? 2. Did these changes make DPP a better fit for your church/community members? Did these changes help participants reach their dietary and physical activity goals? | | | |
| Relative Advantage | 1. Are there any existing lifestyle change programs (nutritional and physical activity programs/resources) available in your community? What are some of the advantages and disadvantages of the DPP sessions compared to these other programs? | | | |
| Cosmopolitanism | 1. Did you develop partnerships with community resources to support DPP at your church? If yes, please describe those partnerships. What has worked well with those partnerships? Have there been any challenges? | | | |
| **Program Members & Communication: Now, we are interested in knowing about your experience working with DPP team members.** | | | | |
| Networks and Communication | 1. How easy or difficult was it to work and communicate with your lifestyle coach and UGA research team to conduct the DPP?   ***Probes***   - How would you describe your communication with these groups? (mode of meetings, frequency, productiveness of meetings) | | | |
| **Resources: Now, we want to know about the resources needed to implement DPP at your site.** | | | |  |
| Access to Knowledge and Information | 1. Did you and your lifestyle coach get sufficient information about carrying out the DPP from the UGA research team?   ***Probes***   - Was the 3-day lifestyle coach training provided to your coaches sufficient for facilitating the DPP sessions? | | | |
| Available Resources | 1. What were the available resources that contributed most significantly to implementing the DPP?   ***Probes***   - What kind of space was used for DPP sessions at your church? Was the space and location suitable for DPP?  1. What do you think of the UGA research team’s support and facilitation to implement DPP?   ***Probes***   - Support regarding funding, information provision, lifestyle coach training  1. What were the most important aspects of this support? (e.g., financial support, planning, resources, information provision, problem-solving, respect/trust). If there were issues, what could be improved? 2. How important is this support for other church (future) sites? 3. What were the biggest resource constraints you encountered while implementing DPP?   ***Probes***   - space, logistics, time | | | |
| Design Quality and Packaging | 1. Did you have sufficient materials (e.g., coach guide, participant handouts, exercise videos) to implement DPP? Could these materials be improved? How? | | | |
| **DPP Participants: Now, we want to ask you some questions about participants’ engagement with the DPP.** | |  | | |
| Engaging: Participants | 1. Did our communication strategy (e.g., recruitment flyers with church name, CDC video to post on the church’s Facebook page) to inform and recruit participants work? If not, what steps should have been taken to encourage the community to participate in DPP? 2. Some individuals from your church or the community may have wanted to participate but did not enroll in the program; what do you think prevented them from participating in the program? (e.g., cost, time constraints, gap in communication, lack of knowledge and motivation, and program format)? 3. Some participants who enrolled in your classes may not complete the DPP sessions; what do you think prevents them from completing the program? (e.g., inconvenient location, lack of transportation to/ from classes) 4. What steps are taken to encourage participants to commit to completing the DPP sessions? What would encourage participants to complete the DPP sessions? | | | |
| Patient Needs and Resources | 1. Has our DPP met the needs and preferences of your church/community members?   ***Probes***   - What kind of impact did the DPP have on participants? What feedback have you received from participants? Any stories of success or failure?  1. Why do you think participants at your church chose to participate in the DPP? (motivation to improve their eating habits, be physically active, prevent diabetes, or improve their overall health) | | | |
| **Reflecting and Evaluating and Recommendations: We are nearing the end of the interview; now, we want to know how you evaluate the success of the DPP and if you think it should be implemented in other church sites.** | | | | |
| Reflecting and Evaluating | 1. Can you describe how the progress of the DPP is being tracked in your church? What measures and sources are you using? (e.g., participation rates, success stories, collecting opinions from participants) | | | |
| Knowledge and Beliefs about the Intervention | 1. What will make the DPP successful/unsuccessful at your church site? How will you measure the success/failure of the program at your church? (weight loss, healthy eating, A1c reduction, participant satisfaction) | | | |
| Available Resources | 1. What resources would be needed to keep the DPP going on a long-term basis in your community? | | | |
| Organizational Incentives & Rewards | 1. What incentives did you receive to conduct the DPP at your church? What incentives would be needed to keep the DPP going? | | | |
| Recommendations | 1. Please provide three recommendations that would increase the likelihood of success of other rural communities to implement DPP sessions? | | | |
| Demographics | 1. Finally, we have some demographic questions:   Please tell us your age, race/ethnicity, gender, and education level. | | | |
| **CLOSING: Those are all the questions we had for you today. Please feel free to add anything we may have missed in this interview.**  **Thank you for taking the time to speak with us today!** | | | |  |

**Lifestyle Coach Interview Guide**

The purpose of this interview is to help us understand factors related to conducting the Diabetes Prevention Program (DPP) sessions in the church setting. During the interview, we will ask you about your opinions of DPP and your experience in carrying out the DPP in your church. Your answers will help us to evaluate the program and improve it for the future.

| **CFIR domain and constructs** | **Interview Questions** | | | |
| --- | --- | --- | --- | --- |
| Role in Church and the DPP | 1. Can you share your roles and responsibilities in your church and the DPP?   ***Probes***   - How did your role as a church member/community member help implement DPP at your church? | | | |
| Program Administration | 1. Can you walk us through how the DPP ran at your church site on a day-to-day basis?   ***Probes***   - CDC recognition process and paperwork, collaboration with your church leadership, participant recruitment/ retention, DPP sessions, data management  1. Can you describe your overall experience related to the DPP? In general, how is the program going? | | | |
| **DPP Implementation and Program Characteristics: Let’s talk about the DPP at your church.** | | |  | |
| Planning | 1. Did you have a clearly communicated implementation plan for the DPP? Can you describe the plan you have and how it helped or hindered implementation?   ***Probes***   - Was it a formal plan? Was it written down somewhere? And was it updated? - What would you suggest for future church sites when planning the DPP implementation? | | | |
| Compatibility | 1. Did the program fit with the church’s mission and the needs of the community? | | | |
| Evidence Strength and Quality | 1. Before starting the program, what did you know about how well DPP works? (For example, how well does the program help with things like eating healthier, losing weight, or lowering HbA1c levels?   ***Probes***   - Where did you get this information from? (e.g., internet, CDC guidelines, UGA research team presentations, your primary care physician, community pharmacy) | | | |
| Complexity | 1. What was the most difficult part of carrying out the DPP in your church/community? 2. What was the easiest part of implementing the DPP? | | | |
| Adaptability | 1. Did you need to make any changes to facilitate the DPP sessions in your church? If so, what changes were made? 2. Did these changes make DPP a better fit for your DPP participants? Did these changes help participants reach their dietary and physical activity goals? | | | |
| Relative Advantage | 1. Are there any existing lifestyle change programs (nutritional and physical activity programs/resources) available in your community? What are some of the advantages and disadvantages of the DPP sessions compared to these other programs? | | | |
| Cosmopolitanism | 1. Did you develop partnerships with community resources to support DPP at your church? If yes, please describe those partnerships. What has worked well with those partnerships? Have there been any challenges? | | | |
| **Program Members & Communication: Now, we are interested in knowing about your experience working with DPP team members.** | | | | |
| Networks and Communication | 1. How easy or difficult was it to work and communicate with your church leadership and UGA research team to conduct the DPP?   ***Probes***   - How would you describe your communication with these groups? (mode of meetings, frequency, productiveness of meetings) | | | |
| **Resources: Now, we want to know about the resources needed to implement DPP at your site.** | | | |  |
| Access to Knowledge and Information | 1. Did you get sufficient information about carrying out the DPP from the UGA research team?  - ***Probes***: Was the 3-day lifestyle coach training sufficient for effectively leading the DPP sessions? | | | |
| Available Resources | 1. What were the available resources that contributed most significantly to implementing the DPP?   ***Probes***   - What kind of space was used for DPP sessions at your church? Was the space & location suitable for DPP?  1. What do you think of the UGA research team’s support and facilitation to implement DPP?   ***Probes:*** Support regarding funding, information provision, lifestyle coach training   1. What were the most important aspects of this support? (e.g., financial support, planning, resources, information provision, problem-solving, respect/trust). If there were issues, what could be improved? 2. How important is this support for other church (future) sites? 3. What were the biggest resource constraints you encountered while implementing DPP?   ***Probes***   - space, logistics, time | | | |
| Design Quality and Packaging | 1. Did you have sufficient materials (e.g., coach guide, participant handouts, exercise videos) to implement DPP? Could these materials be improved? How? | | | |
| **DPP Participants: Now, we want to ask you some questions about participants’ engagement with the DPP.** | |  | | |
| Engaging: Participants | 1. Did our communication strategy (e.g., recruitment flyers with church name, CDC video to post on the church’s Facebook page) to inform and recruit participants work? If not, what steps should have been taken to encourage the community to participate in DPP? 2. Some individuals from your church or the community may have wanted to participate but did not enroll in the program; what do you think prevented them from participating in the program? (e.g., cost, time constraints, gap in communication, lack of knowledge and motivation, and program format)? 3. Some participants who enrolled in your classes may not complete the DPP sessions; what do you think prevents them from completing the program? (e.g., inconvenient location, lack of transportation to/ from classes) 4. What steps are taken to encourage participants to commit to completing the DPP sessions? What would encourage participants to complete the DPP sessions? | | | |
| Patient Needs and Resources | 1. Has our DPP met the needs and preferences of your church/community members?   ***Probes***   - What kind of impact did the DPP have on participants? What feedback have you received from participants? Any stories of success or failure?  1. Why do you think participants at your church chose to participate in the DPP? (motivation to improve their eating habits, be physically active, prevent diabetes, or improve their overall health) | | | |
| **Reflecting and Evaluating and Recommendations: We are nearing the end of the interview; now, we want to know about how you evaluate the success of the DPP and if you think it should be implemented in other church sites.** | | | | |
| Reflecting and Evaluating | 1. Can you describe how the progress of the DPP is being tracked in your church? What measures and sources are you using? (e.g., participation rates, success stories, collecting opinions from participants) | | | |
| Knowledge and Beliefs about the Intervention | 1. What will make the DPP successful at your church site? How will you measure the success/failure of the program at your church? (weight loss, healthy eating, A1c reduction, participant satisfaction) | | | |
| Available Resources | 1. What resources would be needed to keep the DPP going on a long-term basis in your community? | | | |
| Organizational Incentives & Rewards | 1. What incentives did you get to conduct the DPP? What incentives would be needed to keep the DPP going? | | | |
| Recommendations | 1. Please provide three recommendations that would increase the likelihood of success of other rural communities to implement DPP sessions. | | | |
| Demographics | 1. Finally, we have some demographic questions:   Please tell us your age, race/ethnicity, gender, and education level. | | | |
| **CLOSING: Those are all the questions we had for you, today. Please feel free to add anything we may have missed.**  **Thank you for taking time to speak with us today!** | | | |  |
